# F129. COMBINED PATTERNS OF TOBACCO AND CANNABIS USE IN ADOLESCENCE AND THEIR ASSOCIATION WITH PSYCHOTIC EXPERIENCES: A LONGITUDINAL ANALYSIS

**DOI:** 10.1093/schbul/sby017.660

**Published:** 2018-04-01

**Authors:** Stanley Zammit, Hannah Jones, Suzanne Gage, Jon Heron, Matthew Hickman, Glyn Lewis, Marcus Munafo

**Affiliations:** 1 Cardiff University; 2 University of Bristol; 3 University of Liverpool; 4 University College London

## Abstract

**Background:**

There has been increasing concern about potentially causal effects of tobacco use on psychosis, but epidemiological studies have been less robust in attempts to minimise effects of confounding than studies of cannabis use have been. We therefore aim to examine the association of patterns of cigarette and cannabis use with preceding and subsequent psychotic experiences, and compare patterns of confounding across these patterns.

**Methods:**

We analysed repeated measures of cigarette and cannabis use during adolescence in a sample of 5,300 individuals in the Avon Longitudinal Study of Parents and Children birth cohort who had at least 3 measures of cigarette and cannabis use between ages 14–19 years. Cigarette and cannabis use data were summarised using longitudinal latent class analysis to identify longitudinal classes of substance use, and associations between classes and psychotic experiences at 18 years were assessed.

**Results:**

Prior to adjusting for a range of potential confounders, there was strong evidence that early-onset cigarette-only use (4.3%), early-onset cannabis use (3.2%), and late-onset cannabis use (11.9%), but not later-onset cigarette-only use (14.8%) latent classes were associated with increased psychotic experiences compared to non-users (65.9%) (omnibus P<0.001). After adjusting for confounders, the association for early-onset cigarette-only use attenuated substantially (unadjusted odds ratio (OR) = 3.03, 95%CI 1.13, 8.14; adjusted OR = 1.78, 95%CI 0.54, 5.88), whereas those for early-onset (adjusted OR = 3.70, 95%CI 1.66, 8.25) and late-onset (adjusted OR = 2.97, 95%CI 1.63, 5.40) cannabis use were unchanged.

**Discussion:**

Our findings indicate that whilst individuals who use either cannabis or cigarettes during adolescence have an increased risk of developing subsequent psychotic experiences, the epidemiological evidence for this being causal is substantively more robust for cannabis than it is for tobacco

